# Assessment of the Dietary Habits of Women with Fertility Disorders Preparing for In Vitro Fertilization (IVF) and Development of Nutritional Protocol for Women Undergoing IVF

**DOI:** 10.3390/nu18132161

**Published:** 2026-07-03

**Authors:** Małgorzata Szulińska, Shahla Wunderlich, Danuta Gajewska

**Affiliations:** 1 Department of Dietetics, Institute of Human Nutrition Sciences, Warsaw University of Life Sciences (WULS), 159C Nowoursynowska Str., 02-776 Warsaw, Poland; s210425@sggw.edu.pl; 2 Department of Nutrition and Food Studies, Montclair State University, 1 Normal Avenue, Montclair, NJ 07043, USA; wunderlichs@montclair.edu

**Keywords:** infertility, in vitro fertilization (IVF), diet, ProFertiMed score

## Abstract

**Background/Objectives:** Infertility is a multifactorial condition influenced by both medical factors and lifestyle-related determinants, including diet quality and nutritional supplementation. However, it is unclear which dietary patterns are optimal for women requiring assisted reproductive technology (ART). This study had two main objectives: (1) to assess dietary habits and the prevalence of nutritional supplementation among women with fertility disorders preparing for in vitro fertilization (IVF); (2) to develop, based primarily on a review of the scientific literature, a nutritional protocol to support women undergoing assisted reproductive procedures. **Methods:** A cross-sectional study was conducted among 61 women undergoing IVF treatment. Diet quality was assessed using the original ProFertiMed score (food-based score). Data regarding supplementation practices, anthropometric characteristics, and the number of IVF attempts were collected using a structured questionnaire. Statistical analyses (Chi-square test) were performed to evaluate associations between diet quality, supplementation practices, and clinical outcomes. **Results:** Only 8% of the respondents demonstrated a high level of dietary adherence according to the ProFertiMed score, suggesting that nutritional intervention should be implemented in the remaining 92% of women. Dietary supplement use was highly prevalent (95%), with participants taking a mean of 7 ± 4 supplements (range: 1–17). No statistically significant association was observed between diet quality assessed using the ProFertiMed score and the number of IVF attempts (Chi-square test, *p* = 0.85). **Conclusions:** The ProFertiMed score appears to be a promising tool for assessing diet-related factors in the context of assisted reproduction and may be particularly valuable when applied at the time of female infertility diagnosis, allowing for the early identification and modification of dietary factors that may affect reproductive outcomes. The proposed protocol, which is primarily based on the scientific literature, outlines key aspects of supportive nutritional management that may contribute to preconception preparation; however, its potential impact on IVF outcomes has not yet been empirically confirmed and should be evaluated in future studies. Therefore, further refinement and validation are required before its implementation in clinical practice.

## 1. Introduction

Female infertility constitutes a significant public health problem worldwide. According to the definition of the World Health Organization, it is defined as the inability to achieve pregnancy after more than 12 months of regular unprotected sexual intercourse. Globally, infertility affects millions of families, involving approximately one in seven couples in developed countries and one in four couples in developing countries [1]. Infertile couples are increasingly opting for innovative treatment methods such as assisted reproductive technologies (ARTs), including in vitro fertilization (IVF). Despite access to modern techniques for embryo selection, ovarian stimulation, and cryopreservation, IVF is not always effective and does not always result in pregnancy [2].

When analysing the causes of treatment failure, increasing attention is being paid to the impact of improved dietary habits and lifestyle on IVF outcomes [3]. The primary dietary model demonstrating the most beneficial effects is the Mediterranean diet. Evidence suggests that a balanced dietary pattern rich in vegetables, fruits, whole grains, fish, and olive oil may improve ovarian response or oocyte quality [4]. Due to the considerable heterogeneity of studies and potential methodological limitations, there is still no single established dietary model for patients experiencing infertility [5].

In 2025, the World Health Organization issued the latest recommendations on the prevention, diagnosis, and treatment of infertility. In addition to medical aspects, these guidelines emphasize the important role of preconception care, including proper nutrition that provides essential vitamins and minerals. The report highlights the importance of educating patients about a balanced diet and a healthy lifestyle, such as engaging in regular physical activity, smoking cessation, and avoiding alcohol consumption [6].

The aim of the present study was to develop an original assessment tool, the ProFertiMed score, as well as a protocol for women preparing for assisted reproductive procedures, in order to evaluate dietary patterns and the potential impact of diet and lifestyle factors on infertility treatment outcomes.

## 2. Materials and Methods

### 2.1. Study Population

A purposive sampling method was used, including women diagnosed with infertility who were undergoing IVF procedures. The exclusion criteria were age below 18 years and the absence of a diagnosed infertility condition. The questionnaire was distributed among patients of one of the infertility treatment clinics in Warsaw, Poland. Participation in the study was voluntary and anonymous. The study protocol was approved by the Rector’s Committee for Ethics of Research Involving Human Participants at the Warsaw University of Life Sciences (approval no. 16/RKE/2025/U, issued on 2 July 2025).

### 2.2. Questionnaire

We used a structured self-administrated questionnaire designed to collect the important information, relevant in the context of fertility disorders. The tool consisted of three sections and included single-choice and multiple-choice closed-ended questions, as well as open-ended questions. The first section concerned habitual dietary patterns and supplementation use, the second addressed the respondents’ health status and lifestyle, while the third collected demographic data and enabled the assessment of nutritional status based on BMI (Body Mass Index) according to WHO criteria, as well as the characterization of the study group.

The health-related section also included questions concerning infertility, including its diagnosed type, stage of treatment, and the number of IVF attempts. The analysis distinguished the following categories of infertility: primary, secondary, idiopathic, and “other.” The “other” category included responses that did not meet clear classification criteria or indicated a multifactorial etiology, such as immunological factors, age-related infertility, absence of fallopian tubes, ovulatory disorders, or unspecified responses.

### 2.3. Dietary Assessment and Supplement Intake

An abbreviated 11-item food frequency questionnaire (FFQ) was used in the study to estimate habitual food intake of all participants. The questionnaire included questions on the frequency of consumption of selected food groups over the past month and the number of servings consumed. Participants were asked to report how often they had consumed each of the food groups included in the FFQ according to a 6-grade scale (once a month or less often, several times a month, once a week, several times a week, once a day, several times a day). To facilitate accurate reporting, serving sizes (in grams), examples of food products, and their common substitutes were provided.

Adherence to nutritional recommendations regarding the number of servings was evaluated based on criteria established by the National Centre for Nutritional Education (Healthy Eating Plate) [7], as well as the Mediterranean diet model [8,9,10], the DASH diet [11], and the MIND diet [12]. Table 1 specifies the optimal values corresponding to the nutritional recommendations of each model for every food group. Among the analyzed dietary patterns, the DASH diet is the only one providing recommended serving numbers relative to a 2000 kcal intake [13]. The Healthy Eating Plate, the Mediterranean diet, and the MIND diet specify recommended servings but do not define them in relation to specific caloric requirements.

As part of the supplement intake analysis, respondents were asked about specific vitamins and minerals with potential beneficial effects, as well as the name of the supplement and the dosage used [14,15]. Supplement doses were evaluated solely based on the composition of the supplement, not dietary intake. The assessment of supplementation was conducted in accordance with the Polish Nutrition Standards 2024 [16] and recommendations from Polish Scientific Societies [17,18].

### 2.4. Development of the ProFertiMed Score

To assess the quality of diets of women with fertility disorders, a scoring system, the “ProFertiMed score,” was developed. This score synthesizes three indices such as the MedDietScore (Mediterranean Diet Score), the DASH (Dietary Approaches to Stop Hypertension) Diet Score, the MIND Diet Score and recommendations for specific products associated with a pro-fertility diet [19,20]. The components of the scale were selected by the authors based on a review of the scientific literature, current dietary guidelines and additional expert consultations, who evaluated the relevance and clarity of individual items. Due to differences in the number and type of food items assessed across the MedDietScore, DASH Diet Score, and MIND Diet Score, eleven food categories common to all three indices were included in the ProFertiMed score, with the exception of berries, which were added due to their documented beneficial effect on fertility [21].

The score comprises 11 items regarding the number of servings of the following products: whole-grain cereals, vegetables, fruits, berries, milk and dairy products, legumes, red meat, fish, nuts and seeds, olive oil, and alcohol.

These food products are considered beneficial for reproductive health because they reduce inflammation and oxidative stress while improving hormonal and metabolic regulation. Whole-grain cereal products, vegetables, and fruits provide not only essential vitamins and minerals but also dietary fibre. Studies have demonstrated that a high intake of these foods is associated with a greater likelihood of embryo implantation and live birth [22,23].

The consumption of berries, which are a rich source of antioxidants, as well as nuts and seeds, which provide mono- and polyunsaturated fatty acids, was included due to their potential role in reducing oxidative stress and, consequently, their possible beneficial effect on ovarian reserve [21,24]. Legumes, as a major source of plant-based protein, also represent an important dietary component. Replacing 5% of animal protein intake with plant protein may reduce the risk of ovulatory infertility by up to 50%. In addition, plant protein improves insulin sensitivity, thereby exerting a positive effect on carbohydrate metabolism [25].

Fish consumption is also important because of the high content of omega-3 fatty acids, including docosahexaenoic acid (DHA), which may support ovulation and help maintain endometrial receptivity [22,26]. Olive oil should constitute the primary source of dietary fat due to its high polyphenol content and anti-inflammatory properties. Recent studies suggest that olive oil consumption may increase the likelihood of gestational sac detection following embryo transfer [27].

Adequate intake of dairy products is another important component of the diet. It has been suggested that consuming more than three servings of dairy products per day reduces the risk of endometriosis diagnosis by 18% compared with consuming two servings daily. However, findings regarding the fat content of dairy products remain inconsistent. Some studies indicate that low-fat dairy products may increase the risk of ovulatory infertility, whereas high-fat dairy products may enhance fertility; however, other studies have not confirmed such associations [28]. Nevertheless, it appears beneficial to include at least one serving of fermented dairy products in the diet due to their positive effect on gut microbiota, insulin sensitivity, and glucose tolerance [29].

#### Score Calculation and Interpretation

For each of the listed food categories, a score ranging from 0 to 2 points was assigned, where 0 points indicated low intake, 1 point indicated moderate intake, and 2 points indicated high intake of a given product. Detailed scoring criteria for each food category, including serving size definitions and frequency thresholds, are presented in Table 2. Reverse scoring was applied to red meat consumption, where higher values were assigned to lower intake frequencies. Given the negative impact of alcohol on fertility and the reduced probability of conception [30], this variable was assessed separately using a dichotomous scale of 0–2 points (2 points—no consumption; 0 points—any amount of alcohol consumption). A total score was obtained by summing individual item scores, resulting in a possible range from 0 to 22 points. Higher score indicated greater adherence to the recommended consumption of selected food group. The observed score was expressed as a percentage of the maximum possible score:

Percentage Score = (Observed score/22) × 100

Given the limited sample size, score interpretation based on the percentage of the maximum attainable score was preferred over tertile-based categorization. This approach provides stable and reproducible thresholds and allows comparison across studies irrespective to sample-specific score distribution. To answer the question: “Did participants achieve a high level of adherence to dietary recommendations?”, total scores were categorized according to the percentage of the maximum attainable score as: low adherence (<50%, score range 0–10), moderate adherence (50–75%, score range 11–16), and high adherence (>75%, score range 17–22).

Supplementation was not included in the “ProFertiMed score,” as it should be tailored individually to the patient’s needs.

The number of completed IVF cycles was selected as a study variable because it reflects the duration and complexity of a patient’s fertility treatment history. Patients undergoing multiple unsuccessful cycles may represent individuals with prolonged exposure to potentially modifiable lifestyle and nutritional factors, making this group particularly relevant when assessing the role of diet quality in fertility treatment outcomes. The variable “number of IVF attempts” could only be determined for women who had completed at least one treatment cycle (*N* = 50); the remaining participants (*n* = 11) were undergoing ovarian stimulation at the time of the survey and were therefore excluded from analyses involving this variable.

The ProFertiMed score was developed for the purposes of this study and applied for the first time in its current form; therefore, a formal assessment of its reliability (e.g., internal consistency) and validity (e.g., test–retest reproducibility or comparison with existing, validated dietary assessment tools) has not yet been conducted.

### 2.5. Statistical Analysis

Data from the questionnaire were analyzed using Statistica 13.1. (TIBCO Software Inc., Palo Alto, CA, USA). A database was created from the responses. The choice of statistical tests depended on the distribution of the analyzed variables. Normality of distribution was assessed using the Shapiro–Wilk test. Basic statistical parameters were calculated, including mean, median, standard deviation, and lower and upper quartiles. The Chi^2^ test was used for group comparisons. Spearman’s rank correlation coefficient was used to evaluate correlations between ordinal variables and the Mann–Whitney U test was used to compare the dependent variable between two independent groups. A multiple regression analysis was performed to assess the relationship between the number of IVF attempts and the ProFertiMed score, adjusted for age and BMI as potential confounders A significance level of α = 0.05 was adopted for all statistical analyses.

## 3. Results

### 3.1. Characteristics of the Study Group

The study included 61 women attending a fertility clinic in Warsaw, Poland, with a mean age of 35.12 (± 5.28) years. The youngest participant was 19 years old, and the oldest was 45 years. The mean body mass index (BMI) was 23.3 ± 3.7 kg/m^2^ (range: 18.4–36.7 kg/m^2^). All sociodemographic and lifestyle-related data are presented in Table 3.

The largest group consisted of women with primary infertility, followed by those classified as “other” and those with idiopathic infertility. With regard to the stage of treatment, the most numerous subgroups comprised women preparing for cryotransfer or those in the post-procedure phase. Among the declared responses, nearly half of the participants (48%) were undergoing the procedure for the third time or more, highlighting both the scale of the problem and the duration of treatment.

A total of 77% of respondents reported being diagnosed with one or more medical conditions. The most frequently reported conditions were endometriosis, hypothyroidism or hyperthyroidism, polyendocrine metabolic ovarian syndrome (PMOS, formerly polycystic ovary syndrome, PCOS) [31], and insulin resistance. A detailed description of the treatment-related data is presented in Table 4. Statistical analysis did not reveal significant associations between the clinical parameters examined (infertility type, number of IVF attempts, treatment stage, number of medical conditions) or between these variables and respondents’ age and BMI (*p* > 0.05).

**Figure 1:**
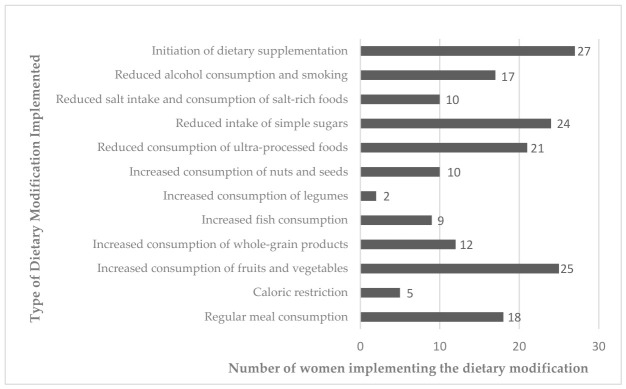
Dietary habits practiced during IVF treatment among women with fertility disorders.

Since the initiation of treatment, 54% of respondents (*n* = 33) reported modifying their dietary habits, whereas 46% (*n* = 28) reported no dietary changes. Among those who modified their diet, the vast majority (91%, *n* = 30) had maintained these changes for several months, while the remaining participants (9%, *n* = 3) had sustained them for only a few weeks. The number of dietary modifications implemented ranged from 1 to 10, with a median of 5. The most frequently reported change was the initiation of dietary supplementation, followed by increased consumption of fruits and vegetables and reduced intake of simple sugars and ultra-processed foods (Figure 1).

### 3.2. Nutrition

Data analysis revealed that 34.4% of participants did not follow any specific diet, 26.2% adhered to an elimination diet (e.g., lactose-free or gluten-free diet), 23% followed a low-glycemic index diet, 11.5% followed an anti-inflammatory diet, and only 4.9% adhered to a reduced-calorie diet. Table 5 presents the intake of specific food groups according to the ProFertiMed score index. The results clearly indicate that the consumption of food groups considered important in a fertility-supporting diet was insufficient. A high intake was observed in more than half of the participants only for vegetables. Whole-grain cereal products, fruits, milk, and dairy products were consumed in moderate amounts, whereas berries, legumes, fish, nuts and seeds, and olive oil were consumed in low amounts. Particular attention should be paid to the consumption of red meat and alcohol. Most respondents reported low red meat intake, which is consistent with the recommendations of the Healthy Eating Plate model. However, in the case of alcohol consumption, a relatively large proportion of participants (41%) declared consuming at least some amount of alcohol. The theoretical maximum score of the scale was 22 points; however, no participant in the study sample obtained a score higher than 18 points. The largest group consisted of women with a low level of adherence to dietary recommendations according to the ProFertiMed score (*n* = 23; 38%). Moderate dietary adherence was observed in 33 women (54%), whereas a high level of adherence was recorded in only 5 women (8%). Spearman’s rank correlation analysis revealed statistically significant associations between adherence to pro-fertility dietary recommendations and frequency of alcohol consumption (rho = −0.385; *p* = 0.002) and body mass index (BMI) (rho = 0.276; *p* = 0.031), *N* = 61 for all analyses. No statistically significant correlation was found with physical activity level (rho = 0.233; *p* = 0.071). The strength of all observed correlations was weak to moderate according to Cohen’s classification.

Diet quality assessment using the ProFertiMed score did not reveal statistically significant differences between groups of women differing in the number of IVF attempts (chi-square test, *p* = 0.85) (Table 6). Regardless of the number of procedures performed, adherence to dietary recommendations was similar across groups. Additionally, no statistically significant differences were found in the ProFertiMed score values depending on the stage of treatment (chi-square test, *p* = 0.76) or the type of diagnosed infertility (chi-square test, *p* = 0.91). A multiple regression analysis was performed to assess the relationship between the number of IVF attempts and the ProFertiMed score, adjusting for age and BMI as potential confounders (*n* = 50; see Methods for details on this subgroup). The overall model was statistically significant [F(3.46) = 3.05; *p* = 0.038], explaining 16.6% of the variance in the ProFertiMed score (adjusted R^2^ = 0.111). However, none of the individual predictors reached statistical significance at the *p* < 0.05 level: number of IVF attempts (β = −0.118; *p* = 0.395), age (β = 0.253; *p* = 0.070), and BMI (β = 0.272; *p* = 0.052), although age and BMI showed a trend approaching statistical significance.

### 3.3. Supplementation

Collected data indicated that 95% of respondents (*n* = 58) reported taking at least one dietary supplement, while only 5% (*n* = 3) did not take any supplements. The number of supplements used varied between 1 and 17 products per participant. The mean number of supplements taken was 7 ± 4 (Table 7). For most of the analyzed micronutrients (vitamin B_6_, vitamin B_12_, vitamin E, vitamin C, magnesium, iron, zinc, selenium, coenzyme Q10), there are no general recommendations for routine supplementation in the general population; their use is primarily advised in cases of deficiency or increased demand. Therefore, the obtained values will be interpreted with reference to the Recommended Dietary Allowances (RDA) for the Polish population [16]. Participants also reported taking inositol, *N*-acetylcysteine, probiotics, iodine, supplements supporting hair growth, calming agents, antioxidants (e.g., berberine, resveratrol, curcumin), and herbal products (e.g., *Vitex agnus-castus*).

No statistically significant correlations were found between the number of supplements used and the ProFertiMed score (rho = 0.045; *p* = 0.242), BMI (rho = −0.048; *p* = 0.711), or the number of IVF attempts (rho = −0.037; *p* = 0.796). Similarly, analysis of individual supplements (*n* = 12) using the Mann–Whitney U test revealed no significant differences in ProFertiMed score, BMI, or number of IVF attempts between women who used a given supplement and those who did not (*p* ranging from 0.6 to 1.0 for all analyzed supplements).

It should be noted that the responses obtained do not allow for a complete reconstruction of the context surrounding the supplementation undertaken by the women surveyed. It cannot be determined with certainty whether a given supplement was prescribed by a physician, recommended by a dietitian, or chosen independently by the participant, nor at what point in time such a decision was made. This lack of precision constitutes a notable interpretative limitation of the present study.

### 3.4. Protocol for Preparing Women for IVF Procedures

**Figure 2:**
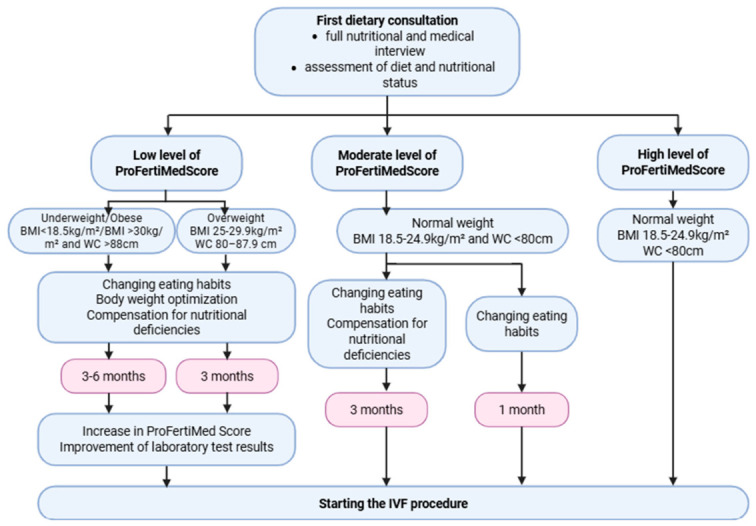
Protocol for preparing women for IVF procedures. BMI—body mass index [kg/m^2^], WC—waist circumference [cm]. Created in BioRender. Szulińska, M. (2026) https://BioRender.com.

The protocol for preparing women for the IVF procedure was developed primarily based on a review of the scientific literature concerning the impact of nutrition on fertility and assisted reproductive treatment outcomes, as well as additional expert consultations. The protocol also incorporates the authors’ own ProFertiMed score as a practical tool for assessing individual’s diet quality and monitoring the progress of the nutritional intervention (Figure 2). Preparation for assisted reproductive procedures should begin with both medical and dietary consultations. A dietitian should conduct a detailed dietary and medical interview and review laboratory test results in order to assess dietary habits, nutritional status, and lifestyle, as well as to identify potential nutrient deficiencies.

An individualized nutrition plan should be developed for each woman undergoing ART treatment (including IVF). Through appropriate nutritional support and improvement of overall health status, such a plan may increase the likelihood of a successful procedure. Preparation should last from 1 to 6 months, depending on the patient’s needs and health status. It is recommended that follow-up consultations with a dietitian take place at least once a month, during which the specialist monitors patient progress, supports behavioral change, and provides nutritional education. It should be noted that, depending on the patient’s medical history, the preparation period for IVF may need to be extended; therefore, the proposed protocol serves as a foundational framework for dietary intervention, which should be further adjusted to individual clinical parameters.

## 4. Discussion

### 4.1. Rationale for the Development of the “ProFertiMed Score” and Management Protocol

The aim of this study was to assess the dietary patterns of women with fertility disorders using the original ProFertiMed score scale and to develop a protocol for women preparing for IVF procedures. At present, there are no detailed dietary guidelines or structured dietary management protocols for this patient population; therefore, an attempt was made to develop such a framework.

Among existing dietary assessment tools used in the context of fertility treatment, the Fertility Diet (FD), associated with a lower risk of ovulatory infertility [32], and the Profertility Diet (PFD), whose high adherence was associated with improved IVF outcomes (implantation, clinical pregnancy, live birth), are particularly noteworthy [3]. Another prospective cohort study also demonstrated an association between adherence to the Mediterranean diet and the success of IVF procedures [19]. Unlike these tools, which are based on a single dietary pattern derived through principal component analysis of detailed food frequency questionnaires (e.g., comprising 131 food items), the ProFertiMed score represents a synthesis of several established and validated dietary indices (MedDietScore, DASH, MIND) combined with fertility-specific dietary recommendations, formulated as a concise, 11-item scoring tool. This design makes the scale more practical and easier to apply in routine dietetic practice compared to the more complex statistical methods required to derive dietary patterns in existing tools.

In designing the protocol, the preconception period was taken into account. During this time, both partners should undergo necessary medical examinations and take care of their health status. Above all, they should adopt a balanced diet and engage in regular physical activity, optimize body weight, address vitamin and mineral deficiencies, introduce appropriate supplementation when needed, and eliminate alcohol consumption and tobacco use, while also taking care of mental health [33,34]. The latest WHO guidelines recommend that the preconception period should last 3 to 6 months, depending on individual needs and the diagnosed cause of infertility [6]. In cases where body weight optimization is required prior to treatment, gradual and safe weight reduction is recommended [35], as very-low-calorie diets aimed at rapid weight loss may even negatively affect treatment outcomes [36]. It is also crucial to correct nutrient deficiencies through a balanced diet and/or supplementation. Depending on the specific nutrient, the time required for correction may range from several weeks to several months, which was also considered in the protocol [37,38,39]. A recent systematic review confirms the necessity of implementing routine assessment of patients’ nutritional status prior to initiating assisted reproductive procedures, as well as the importance of modifying dietary habits to improve the likelihood of successful in vitro fertilization (IVF). The intervention programs lasted between 3 and 6 months and focused on the adoption of a Mediterranean dietary pattern, supplementation, probiotic therapy, or broader improvements in dietary behaviors and reduction in substance use among couples. The findings consistently demonstrated improvements in embryonic morphokinetic parameters, reductions in endometrial inflammatory markers, enhanced intestinal barrier integrity, as well as improved gamete quality and endometrial receptivity [40].

In the present study, no statistically significant association was found between diet quality assessed using the ProFertiMed score and the number of IVF attempts (*p* = 0.85). The results of the multiple regression analysis confirm that the number of IVF attempts was not significantly associated with diet quality, even after adjusting for age and BMI as potential confounders, consistent with the earlier univariate analyses. Although the overall model was statistically significant, this appeared to be driven primarily by the borderline trends for age and BMI rather than by the main variable of interest. This suggests that other unexamined factors may have a stronger influence on diet quality among women preparing for IVF than the experience of previous unsuccessful attempts itself. Systematic reviews suggest that fertility-oriented dietary patterns and the Dutch “preconception diet” are associated with improved outcomes such as biochemical pregnancy, clinical pregnancy, and live birth; however, these findings are based on relatively small study populations. In the case of the Mediterranean diet, the results are inconclusive, and authors emphasize the heterogeneity of the studied populations as well as multiple potential confounding factors, such as the partner’s diet or the number of previous assisted reproductive technology (ART) attempts. Despite this, the Mediterranean diet is still considered one of the main beneficial dietary models [5]. Although the evidence regarding specific dietary patterns is inconsistent, the effects of individual nutrients are well documented. It is known that components such as polyunsaturated fatty acids (PUFAs), folic acid, antioxidants, plant-based proteins, and dietary fiber have beneficial effects, whereas highly processed foods, simple sugars, and trans fats exert adverse effects [41]. Specifically designed predictive models indicate that reproductive potential in women of reproductive age can be improved through inflammation reduction, nutritional optimization, and control of metabolic disorders, further supporting the rationale for dietary and lifestyle modification in person undergoing fertility treatment [42].

In our study, only a small proportion of respondents demonstrated high level of adherence to dietary recommendations. These findings are relevant because an increasing number of studies indicate an association between dietary habits and the effectiveness of infertility treatment. Greater adherence to a healthy dietary pattern or implementation of a supplemented pro-fertility diet was associated with a higher number of retrieved oocytes, an increased proportion of high-quality (Grade A) embryos, and a higher fertilization rate compared with the control group. These findings suggest that appropriate dietary modifications and supplementation may represent safe, feasible, and cost-effective supportive strategies for optimizing embryological outcomes in assisted reproductive technology (ART) procedures [42,43].

It should be emphasized that the ProFertiMed score is a pilot tool and requires further validation and application in studies involving larger populations. Future work should focus on its refinement and validation in relation to more detailed clinical parameters and infertility treatment outcomes.

### 4.2. The Role of Nutrition

Our study revealed that, following treatment initiation, the majority of respondents reported making changes to their dietary habits, primarily by increasing their consumption of fruits and vegetables and reducing their intake of simple sugars and ultra-processed foods. Numerous studies highlight the benefits of a balanced diet, optimal body weight, and avoidance of stimulants in the context of infertility treatment [44]. Evidence suggests improvements particularly in oocyte quality, embryo implantation rates, and the probability of live birth [45]. Pilot studies also indicate that preconception weight reduction in women with obesity and infertility increases the likelihood of achieving pregnancy, both through IVF and spontaneously [46].

In the presented study, attention was focused exclusively on health-promoting dietary components, without accounting for the intake of ultra-processed foods (UPFs), which may have influenced the study outcomes. High consumption of ultra-processed products is strongly associated with reduced fertility potential. A population-based study of 2582 American women of reproductive age (20–45 years) showed that each 10-percentage-point increase in the proportion of UPFs in the diet was associated with a significant decline in fertility [47]. Another prospective cohort study including 831 women demonstrated that higher maternal UPF consumption was associated with reduced early embryonic growth and smaller yolk sac volume during the first trimester of pregnancy, further highlighting the importance of overall dietary quality in the preconception period [48].

A Mediterranean-style diet enriched with key nutrients such as folate, iron, vitamin D, calcium, zinc, vitamin B_12_, and long-chain omega-3 fatty acids (DHA) supports hormonal regulation, ovulation, proper pregnancy progression, and reduces the risk of chronic diseases [49]. Folic acid, through activation of the maturation-promoting factor (MPF) via the Mos–MEK–MAPK–RSK signaling pathway, may increase the number of oocytes and embryos. Antioxidants help mitigate oxidative damage, while alpha-linolenic acid serves as a precursor of prostaglandins, which play a key role in maintaining endometrial receptivity and may facilitate embryo implantation. Additionally, vitamin B_6_ (similar to folic acid) has been associated with a potential improvement in ovarian response and oocyte quality [4].

### 4.3. The Role of Supplementation

In our study, contrary to expectations, no significant differences in supplementation patterns were observed according to adherence to the ProFertiMed score, BMI, or fertility treatment history. These findings are challenging to interpret. However, it cannot be excluded that supplementation practices among the studied women were not fully individualized to their nutritional or metabolic profiles, but rather reflected general medical recommendations or widely available information, regardless of individual needs. This observation highlights the potential role of dietitians in tailoring supplementation recommendations as part of IVF preparation, thereby moving beyond standardized, non-individualized approaches.

In the Polish population, routine supplementation with vitamin D and folic acid is recommended due to the high prevalence of deficiency and the prevention of neural tube defects, respectively [17,18]. Current guidelines recommend 1000–2000 IU of vitamin D daily, alongside 400 µg of folic acid and 400 µg of active folate (5-MTHF) for women planning pregnancy. In the present study, 92% of respondents used vitamin D within the recommended range, although instances of excessive intake (e.g., 20,000 IU) were also observed. Regarding folic acid, 11% of respondents reported no supplementation, and only 39% used the more bioavailable methylated form; a trend toward higher 5-MTHF use among women with more IVF attempts did not reach statistical significance (*p* = 0.052). While folate is well established in reducing congenital anomalies and pregnancy complications, its direct impact on assisted reproduction outcomes remains inconclusive [50,51]. Currently, there are no universal supplementation protocols applicable to all women attempting to conceive, either naturally or through assisted reproductive technologies. However, increasing evidence points to potential benefits of selected nutrients. Among the most extensively studied are omega-3 fatty acids. In the present study, 74% of respondents reported DHA supplementation, with a median intake of 276.5 mg/day, corresponding to minimum recommendations. As guidelines refer to combined EPA and DHA intake (≥250 mg/day), total intake may have been higher. Omega-3 fatty acids may improve oocyte quality and modulate the endometrial environment, potentially supporting embryo development [14,45]. Nevertheless, dietary intake—particularly through regular consumption of fatty fish—should remain the primary source [16].

Vitamin B_12_ status and homocysteine levels are also relevant. Elevated homocysteine levels have been associated with impaired embryo development, particularly in frozen embryo transfer cycles. Higher serum vitamin B_12_ levels may correlate with an increased likelihood of live birth in IVF patients [52]. Therefore, monitoring these parameters in both partners prior to treatment appears justified.

Other supplements, including coenzyme Q10, resveratrol, antioxidant vitamins (C and E), myo-inositol, and selected herbal preparations, have also been investigated. Coenzyme Q10 may improve mitochondrial function and ovarian response, while myo-inositol is particularly beneficial in women with PMOS, supporting ovulation and oocyte quality. Evidence for other compounds remains promising but limited and requires further research [53,54,55]. Thus, priority should be given to optimizing diet and lifestyle, with supplementation introduced as a secondary, targeted intervention.

The findings indicate that respondents frequently used multiple supplements, both single-ingredient and multicomponent formulations. Some studies suggest that multicomponent supplementation may improve selected infertility treatment outcomes, including oocyte and embryo quality and clinical pregnancy rates [14,56]. However, the available evidence is heterogeneous and inconclusive. Additionally, duplication of ingredients across different supplements (e.g., folic acid, vitamin D, magnesium) was observed, which may lead to excessive intake. At the same time, most dietary supplements used in female infertility contain ingredients with no proven efficacy or in doses too low to exert a therapeutic effect; therefore, these findings raise serious concerns regarding the effectiveness of many commercially available fertility-supporting products [57]. Study limitations include the lack of access to complete medical records and the self-reported nature of supplementation data, which precludes assessment of appropriateness and supervision.

### 4.4. Health Status and Treatment Process

The etiology of infertility is multifactorial, encompassing both lifestyle-related factors and comorbid conditions. In the present study, the most commonly reported conditions potentially affecting fertility were endometriosis, thyroid disorders, polyendocrine metabolic ovarian syndrome (PMOS), and insulin resistance.

Endometriosis, a chronic inflammatory condition with a hormonal basis, may impair fertility through inflammatory dysregulation, pelvic anatomical changes, and reduced ovarian reserve. It affects up to 10% of women of reproductive age. In this study, 39% of respondents reported a diagnosis, likely reflecting the characteristics of the study population. Mechanisms include endometriomas reducing antral follicle count and anti-Müllerian hormone (AMH) levels, adhesions impairing oocyte transport, and chronic inflammation affecting embryo quality. Lifestyle interventions, including anti-inflammatory dietary patterns and appropriate supplementation, may support symptom management [58,59,60].

Thyroid dysfunction, both hypothyroidism and hyperthyroidism, can disrupt the hypothalamic–pituitary–ovarian axis, affecting gonadotropin secretion and ovulation. In this study, 39% of respondents reported thyroid disorders. Evidence suggests an increased prevalence of infertility among women with Graves’ disease and Hashimoto’s thyroiditis. Lifestyle factors such as maintaining a healthy weight, stress reduction, and avoiding stimulants may support treatment [61,62,63].

PMOS is one of the most common endocrine–metabolic disorders and a leading cause of anovulatory infertility. Its prevalence ranges from 9% to 18%, while in this study, it was 31%. Pathophysiology includes hyperandrogenism and disrupted gonadotropin secretion, leading to impaired follicular maturation and chronic anovulation. It is often associated with insulin resistance, further exacerbating hormonal imbalance. Lifestyle interventions, including weight reduction, balanced diet, and physical activity, may improve metabolic parameters and increase the likelihood of ovulation and pregnancy [64,65,66].

Insulin resistance, characterized by decreased tissue sensitivity to insulin and compensatory hyperinsulinemia, may impair reproductive function by increasing ovarian androgen production. It was reported in 28% of participants, despite a mean BMI of 23.3, indicating its presence even in normal-weight individuals. Excess insulin may exacerbate hyperandrogenism and PMOS-related disturbances [65,67,68].

Although no statistically significant associations were found between these conditions and infertility type or number of IVF attempts, the findings suggest a potential indirect role. Lifestyle factors, including diet and physical activity, may influence disease progression and thereby indirectly affect treatment outcomes. Further prospective studies are required.

### 4.5. Strengths and Limitations

A strength of this study is the focus on whole dietary assessment. We acknowledge that the main limitation of our study is the relatively small number of participants, which may affect the statistical power and the generalizability of our findings to the broader population. Furthermore, due to the cross-sectional design of the study, no causal inferences can be drawn from the results; only potential associations between the analyzed variables could be assessed. Future longitudinal studies are needed to confirm these findings and establish causal relationships. Additionally, the developed model refers exclusively to women; therefore, in future research, both the ProFertiMed score and the preparation protocol for assisted reproductive procedures should be extended to include men, as infertility treatment should be approached at the couple level. Our finding cannot be generalized to the whole reproductive population. In addition, the declarative and estimated nature of dietary data, including supplement use, may influence the accuracy of the results.

Another limitation of this study is that the ProFertiMed score is a newly developed tool that has not yet undergone formal validation. Further research is needed to assess its internal consistency, test–retest reliability, and convergent validity with other established dietary quality indices before it can be recommended for broader clinical use.

Despite these limitations, the study provides important insights into the dietary patterns of women undergoing infertility treatment. This topic remains relatively underexplored, particularly in the Polish population, highlighting the need for further research in this area.

## 5. Conclusions

Our research supports the hypothesis that behavioral intervention should constitute the first step in assisted reproductive technology (ART) protocols, including in vitro fertilization (IVF). Subsequently, an individualized nutrition plan should be developed for each woman undergoing ART treatment. Although the optimal dietary pattern for women requiring ART remains unclear, we believe that the components of the ProFertiMed score may exert synergistic effects on various aspects of fertility and assisted reproduction outcomes. Nevertheless, the ProFertiMed score requires further validation in larger and more diverse populations before its clinical applicability and utility can be established.

## Figures and Tables

**Table 1:** Recommended servings of food groups according to the Mediterranean diet, DASH diet, MIND diet, and Polish dietary guidelines [7,8,9,10,11,12].

Food Groups *	MedDietScore (Servings/Week)	DASH Diet Score (Servings/Day)	MIND Diet Score (Servings/Week)	Polish Standards (Servings/Day)
Whole grains	>31	>6.5 total grains	≥21	>3
Vegetables	>32	>3.5	≥7 green leafy vegetables ≥7 other vegetables	>3
Fruits	>21	>3.5	Not included	>2
Berries	Not included	Not included	≥5	Not included
Milk and dairy products	≤10 full-fat cheese	>2	≤2 full-fat cheese	3 to 4
Fish	>6	≤2	≥1	>1 to 2/per week
Red meat	≤1		<4 meals	<2 to 3/per week
Poultry	≤3		≥2	Not included
Legumes	>6	>3.5/per week	≥3	Not included
Nuts and seeds	Not included		≥5	1
Olive oil	>5	≤27.5% kcal from fat ≤6% kcal from saturated fat	≥14 tablespoons ≤7 pats/week butter and stick margarine	>2 to 3
Alcohol	<300 (mL/day)	Not included	2–7 glasses of wine	0
Sodium	Not included	≤2.400 mg	Not included	≤2000 mg
Fast and Fried Foods	Not included	Not included	≤1 meal	Not included
Pastries & sweets	Not included	≤5.5/per week	<5	Not included

* The table presents values that meet the optimal dietary recommendations.

**Table 2:** Components of the ProFertiMed score.

Food Categories (Servings/Day) *		Score	
	0	1	2
Whole grains (1 serving: 30–40 g)	0 to 1	>1 to 3	≥4
Vegetables ** (1 serving: 80 g)	0 to 1	>1 to 3	≥4
Berries (1 serving: 80 g)	0	-	≥1
Other Fruits (1 serving: 80 g)	0	1 to 2	≥3
Milk and dairy products *** (1 serving: 200 g)	0	1 to 2	≥3
Fish (servings/week) (1 serving: 120 g)	0	1	≥2
Red meat (servings/week) (1 serving: 100 g)	≥3	1 to 2	0
Legumes (servings/week) (1 serving: 50 g raw)	0	1 to 2	≥3
Nuts and seeds (1 serving: 30 g)	0	-	≥1
Olive oil (1 serving: 10 g)	0	1 to 2	≥3
Alcohol (in any form)	≥1	-	0

* The developed index refers to a diet containing approximately 2000 kcal. ** at least 2 recommended servings of leafy vegetables. *** at least 1 recommended serving of fermented dairy products.

**Table 3:** Characteristics of the study population (sociodemographic, health, and lifestyle data) (*n* = 61).

Variable		Frequency, *n* (%)
Age (age distribution ranged exclusively between 19 and 45 years)	19–29	7 (11.5)
	30–34	19 (31.1)
	35–40	25 (41)
	40–45	10 (16.4)
BMI (Body Mass Index)	Underweight	1 (1.6)
	Normal	43 (70.5)
	Overweight	14 (23)
	Obesity	3 (4.9)
Place of residence	Village	9 (14.8)
	Small town	3 (4.9)
	Medium-sized town	7 (11.5)
	Large city	42 (68.9)
Physical activity level	Very low	3 (5)
	Low	16 (26)
	Moderate	34 (56)
	High	7 (12)
	Very high	1 (2)
Cigarette smoking	Yes	1 (2)
	Occasionally	3 (5)
	Very rarely	2 (3)
	No	55 (90)
Frequency of alcohol consumption	3–6 times per week	5 (8)
	1–2 times per week	7 (12)
	2–3 times per month	17 (28)
	Once a month or less	32 (52)
Alcohol servings per week	0 (abstinent)	36 (59)
	1–2 servings	21(34)
	3–6 servings	3 (5)
	7–12 servings	1 (2)
Daily stress level	Low	12 (19)
	Moderate	31 (51)
	High	18 (30)
Stress-inducing factors	Occupational work	32 (53)
	Treatment process	17 (28)
	Social relationships	10 (16)
	Other	2 (3)

**Table 4:** Characteristics of the study group regarding the IVF treatment process.

Variable		Frequency, *n* (%)
Type of infertility	Primary infertility	20 (32.8)
	Secondary infertility	7 (11.5)
	Idiopathic infertility	14 (22.9)
	Male factor infertility	3 (4.9)
	Other	17 (27.9)
Stage of treatment	Undergoing diagnostic evaluation	12 (19.7)
	During ovarian stimulation	8 (13.1)
	Prior to cryotransfer	18 (29.5)
	Post-transfer/post-cryotransfer	23 (37.7)
Number of IVF (in vitro fertilization) attempts	First attempt	14 (28)
	Second attempt	12 (24)
	Third attempt or more	24 (48)
Medical conditions	Endometriosis	24 (39)
	Hypothyroidism/Hyperthyroidism	24 (39)
	Polyendocrine Metabolic Ovarian Syndrome (PMOS)	19 (31)
	Insulin resistance	17 (28)

**Table 5:** Distribution of women across low, moderate, and high adherence to dietary recommendations categories for individual food groups based on the ProFertiMed score index (*n* = 61).

Food Groups	Low Adherence	Moderate Adherence	High Adherence
Whole grains	3 (5%)	35 (57%)	23 (38%)
Vegetables	1 (2%)	23 (38%)	37 (60%)
Fruits	12 (20%)	38 (62%)	11 (18%)
Berries	31 (51%)	0	30 (49%)
Milk and dairy products	15 (25%)	37 (60%)	9 (15%)
Legumes	31 (51%)	8 (13%)	22 (36%)
Red meat	32 (53%)	22 (36%)	7 (11%)
Fish	32 (52%)	18 (30%)	11 (18%)
Nuts and seeds	35 (57%)	0	26 (43%)
Olive oil	37 (60%)	20 (33%)	4 (7%)
Alcohol	36 (59%)	0	25 (41%)

**Table 6:** Adherence to dietary recommendations according to the ProFertiMed score among women by number of IVF attempts.

ProFertiMed Score Level (*n* = 50)	First IVF Attempt	Second IVF Attempt	Third IVF Attempt	*p*-Value *
Low level (0–10 points)	5 (35.7%)	4 (33%)	10 (41.7%)	0.85
Moderate level (11–16 points)	7 (50%)	7 (58%)	13 (54.1%)	
High level (17–22 points)	2 (14.3%)	1 (9%)	1 (4.2%)	

* The *p*-value was calculated using the chi-square test.

**Table 7:** Characteristics of dietary supplements used by study participants.

Supplement	*N* (%)	Mean Daily Dose (SD)	Median Daily Dose	Minimum Dose	Maximum Dose	Daily Recommendations for the Polish Population *
Folic acid		675 µg (±1132)	400 µg	400 µg	8500 µg	400–800 µg [18]
− total − methylated form	54 (89%)					
	24 (39%)					
Vitamin B_6_	34 (56%)	24 mg (±48.5)	5 mg	1.4 mg	250 mg	1.3 mg (RDA)
Vitamin B_12_	34 (56%)	369 µg (±978)	6 µg	2.5 µg	5000 µg	2.4 µg (RDA)
Vitamin D	56 (92%)	3476 IU (±3160)	2000 IU	200 IU	20,000 IU	1000–2000 IU [17]
Vitamin E	17 (28%)	140 mg (±140)	150 mg	3.6 mg	400 mg	8 mg (AI)
Vitamin C	28 (46%)	587 mg (±590)	400 mg	40 mg	2000 mg	75 mg (RDA)
Magnesium	34 (56%)	193 mg (±148)	125 mg	48 mg	600 mg	310–320 mg (RDA)
Iron	14 (23%)	67 mg (±65.5)	40 mg	10 mg	200 mg	18 mg (RDA)
Zinc	24 (39%)	15 mg (±12)	14 mg	5 mg	60 mg	8 mg (RDA)
Selenium	15 (25%)	126 µg (±76)	100 µg	10 µg	200 µg	55 µg (RDA)
Coenzyme Q10	29 (48%)	212 mg (±226)	100 mg	25 mg	1000 mg	No specific recommendations available
Omega-3 (DHA)	45 (74%)	378 mg (±276.5)	276.5 mg	60 mg	1540 mg	250 mg EPA + DHA (RDA)

* Recommendations based on the Polish Nutrition Standards [16].

## Data Availability

The data presented in this study might be available on request from the corresponding author due to ethical reason.
